# Supplementary material for: A rapid aureochrome opto-switch enables diatom acclimation to dynamic light
Source: Nat Commun. 2024 Jul 3;15:5578. doi: 10.1038/s41467-024-49991-7 (PMC11219949; doi:10.1038/s41467-024-49991-7)
Supplement: Supplementary file 1 — Supplementary Information [file 41467_2024_49991_MOESM1_ESM.pdf]

Supplementary information for

A rapid aureochrome opto-switch enables diatom acclimation to dynamic light

Huan Zhang, Xiaofeng Xiong, Kangning Guo, Mengyuan Zheng, Tianjun Cao, YuqingYang, Jiaojiao Song, Jie Cen, Jiahuan Zhang, Yanyou Jiang, Shan Feng, Lijin Tian, Xiaobo Li✉

✉email: lixiaobo@westlake.edu.cn

This file contains:

Supplementary Tables 1-2

Supplementary Figs. 1-21

The **Supplementary Data** Sets are provided as spreadsheets in an Excel file separate from this combined file.

Supplementary Table 1. Pigment composition under growth light (GL) and after 6 h or 1 d of very high light (VHL; 1200 μmol photons m^{-2} s^{-1}) treatment. Car, carotenoid; Ddx, diadinoxanthin; Dtx, diatoxanthin; Fx, fucoxanthin; n.d., not detectable. "DES" stands for "de-epoxidation state", which was calculated as the ratio of Dtx to the sum of Ddx and Dtx. Three independent cultures were used for the measurements. The experiment was repeated twice independently with consistent results and a representative result is shown.

Samples	GL			VHL, 6 h			VHL, 1 d		
	WT	*aureo1c-1*	COMP	WT	*aureo1c-1*	COMP	WT	*aureo1c-1*	COMP
Fx (fg/cell)	789 ± 25	943 ± 33	868 ± 35	699 ± 46	761 ± 9	740 ± 18	224 ± 8	24 ± 8	239 ± 9
Chl c_1 (fg/cell)	106.0 ± 4.6	123.1 ± 3.7	117.4 ± 5.3	103.0 ± 6.2	113.9 ± 3.7	107.7 ± 9.1	35.7 ± 1.2	3.4 ± 2.4	38.7 ± 3.5
Chl c_2 (fg/cell)	110.9 ± 4.1	141.0 ± 5.3	121.5 ± 5.4	96.6 ± 4.9	109.8 ± 2.2	100.7 ± 1.2	23.3 ± 1.0	1.8 ± 1.1	26.7 ± 2.3
Ddx (fg/cell)	123.3 ± 10.8	127.8 ± 2.5	128.5 ± 6.3	64.4 ± 3.0	20.9 ± 3.3	93.4 ± 8.2	16.6 ± 0.7	1.4 ± 1.1	53.8 ± 4.4
Dtx (fg/cell)	3.8 ± 0.4	4.4 ± 0.4	3.8 ± 0.2	103.0 ± 14.3	137.9 ± 7.7	79.0 ± 5.7	52.3 ± 3.2	1.9 ± 1.1	47.1 ± 4.4
Chl *a* (fg/cell)	806 ± 25	938 ± 33	882 ± 35	755 ± 46	821 ± 9	783 ± 18	105 ± 8	15 ± 9	103 ± 9
β-car (fg/cell)	58.0 ± 1.3	63.4 ± 2.7	60.2 ± 2.0	57.6 ± 2.1	65.8 ± 1.2	52.3 ± 2.8	3.4 ± 0.3	n.d.	4.7 ± 1.1
DES	0.031 ± 0.005	0.035 ± 0.003	0.030 ± 0.001	0.620 ± 0.042	0.872 ± 0.013	0.465 ± 0.020	0.764 ± 0.004	0.592 ± 0.113	0.474 ± 0.025

Supplementary Table 2. The content of fucoxanthin (Fx), diadinoxanthin (Ddx) and diatoxanthin (Dtx) after 10-min and 2-day of 900 μmol photons m^{-2} s^{-1} of high light treatment. Three independent cultures were used for the quantification. Standard deviations are provided. Three independent cultures were used for the measurements. The experiment was repeated twice independently with consistent results and a representative result is shown.

Samples	10 min			2 d		
	WT	*aureo1c-1*	COMP	WT	*aureo1c-1*	COMP
Fx (fg/cell)	599 ± 28	697 ± 68	612 ± 29	129 ± 12	83 ± 23	152 ± 9
Ddx (fg/cell)	90.8 ± 5.5	98.6 ± 11.2	94.5 ± 4.9	41.2 ± 4.6	7.3 ± 2.1	68.5 ± 5.4
Dtx (fg/cell)	6.7 ± 0.4	8.6 ± 0.8	7.7 ± 0.5	34.4 ± 3.1	16.2 ± 4.6	25.3 ± 9.2
Chl *a* (fg/cell)	624 ± 22	678 ± 72	633 ± 31	99 ± 11	82 ± 25	139 ± 50
Ddx/Chl *a*	0.223 ± 0.006	0.223 ± 0.006	0.229 ± 0.001	0.637 ± 0.037	0.136 ± 0.009	0.810 ± 0.247
Dtx/Chl *a*	0.0169 ± 0.0014	0.0199 ± 0.0003	0.0193 ± 0.003	0.548 ± 0.014	0.312 ± 0.050	0.287 ± 0.032
DES	0.071 ± 0.007	0.082 ± 0.001	0.078 ± 0.001	0.463 ± 0.018	0.695 ± 0.019	0.272 ± 0.074

Supplementary Figures

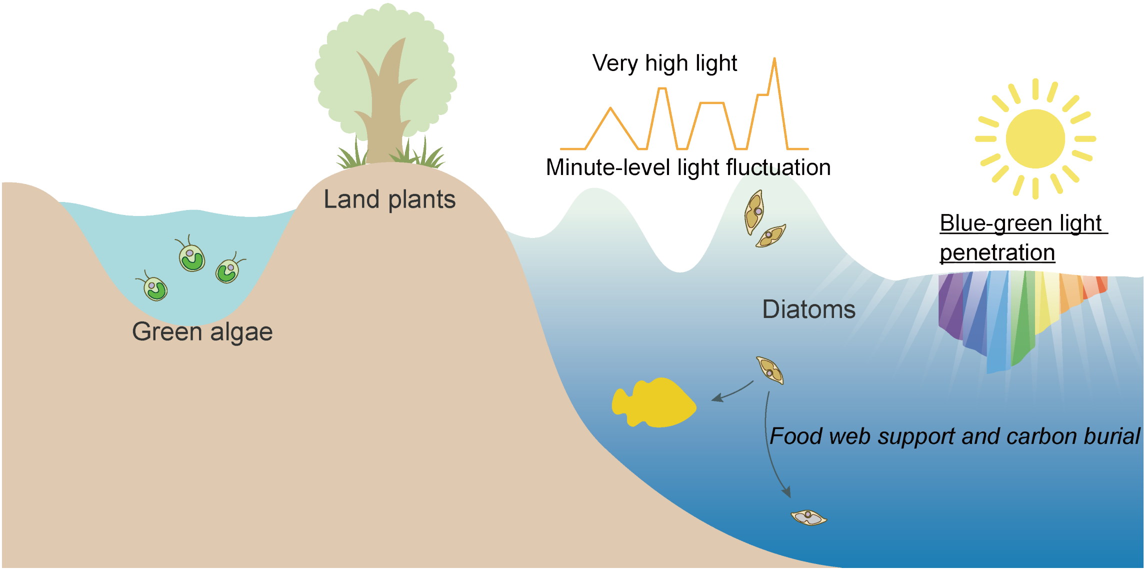

Supplementary Fig. 1 | Importance of light dynamics and photoprotective responses in the biogeography of photosynthetic eukaryotes. More species of green algae live in freshwater or soil environments than in the oceans[1]. Diatoms are the most abundant eukaryotic algae in marine environments and they are abundant in freshwater bodies as well[1]. Diatoms living in highly dynamic estuary or coastal environments with dynamic light conditions[24,25,31] survive fluctuating light better than green algae[29]. Please refer to reference list of the main text.

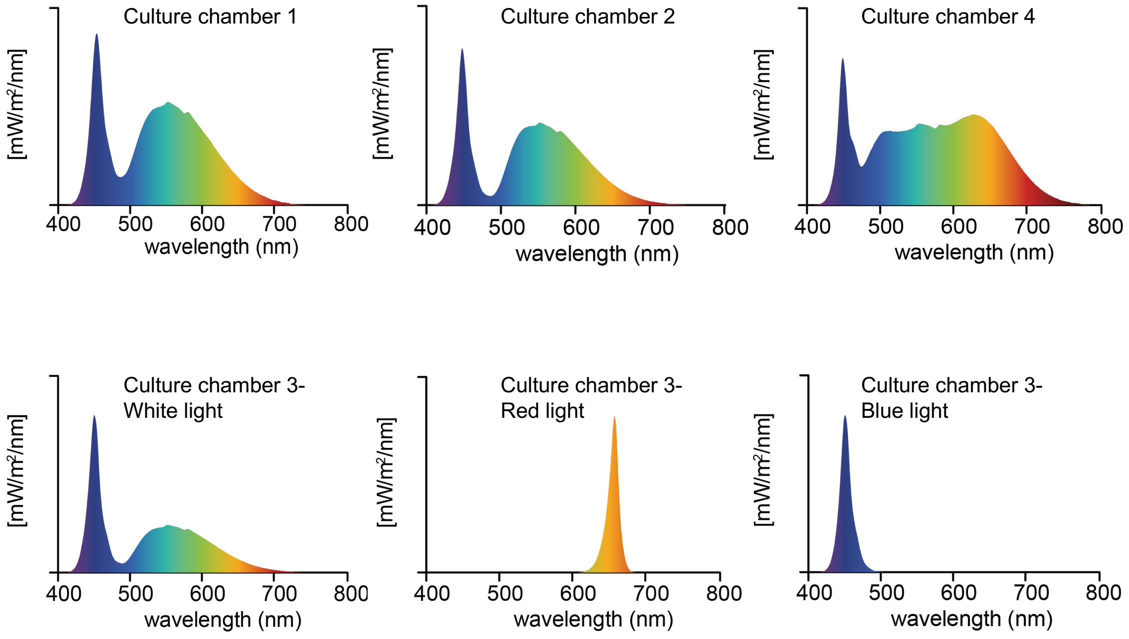

Supplementary Fig. 2 | The light spectrum of each culture chamber. For each experiment, the light intensity and quality as well as the culture chamber used are indicated in **Supplementary Data 2.**

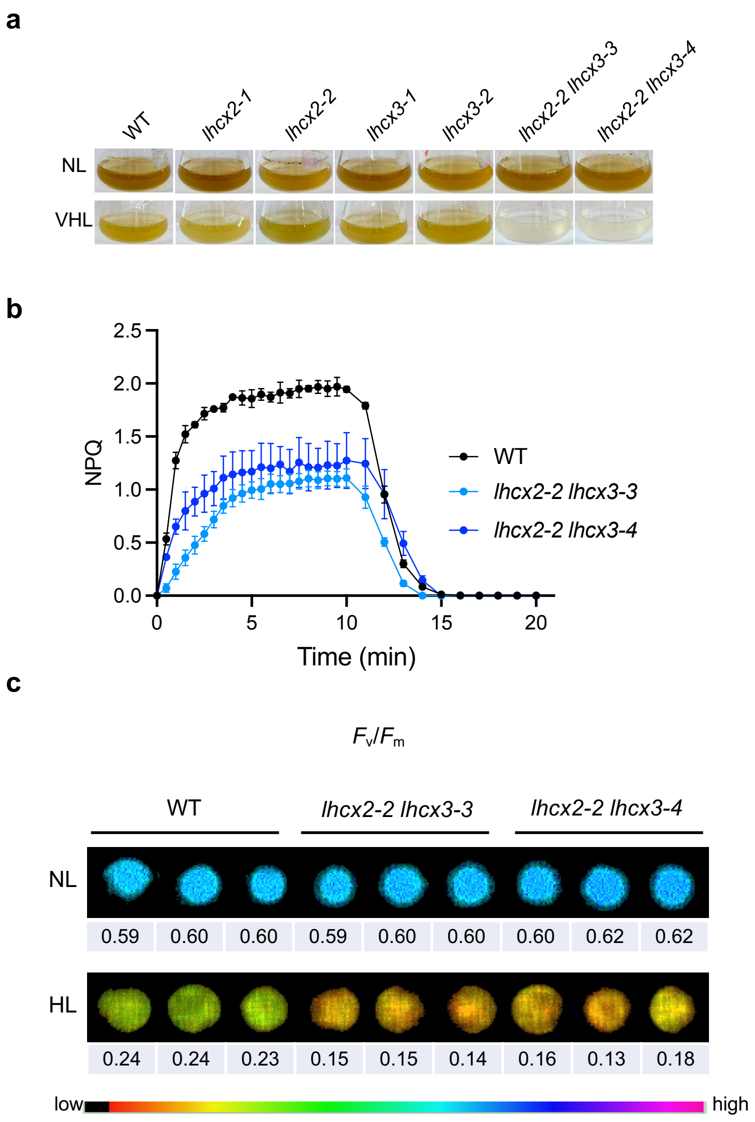

Supplementary Fig. 3 | The *P. tricornutum* photoprotection effectors LHCX2 and LHCX3 are required for cell fitness under high light. a, Photographs of growth flasks of the wild type (WT), single and double mutants deficient in LHCX2 and/or LHCX3 function, after 2 days of growth under growth light (GL) or very high light (VHL; 1200 µmol photons m^{-2} s^{-1}). **b,** Non-photochemical quenching (NPQ) phenotypes of WT and *lhcx2 lhcx3* double mutants after 6 hours of growth under 900 µmol photons m^{-2} s^{-1} of white light. Three independent cultures were used for the quantification. Data are presented as mean values ± standard deviations (SD). **c,** Maximal quantum yields of

photosystem II (PSII) of *lhcx2 lhcx3* double mutants, after growth under growth light (GL; 40 µmol photons m^{-2} s^{-1} of white light) and 3 days of treatment under high light (HL; 550 µmol photons m^{-2} s^{-1} of white light), measured as the ratio between variable chlorophyll fluorescence (F_v) and maximum fluorescence (F_m) after dark acclimation (see **Methods**). The F_v/F_m ratios of three independent cultures are shown as false-color images in addition to the values. For panels **a, b** and **c,** the experiment was repeated three times independently with similar results and a representative result is shown. Source data are provided as a Source Data file.

Supplementary Fig. 4 (to be continued)

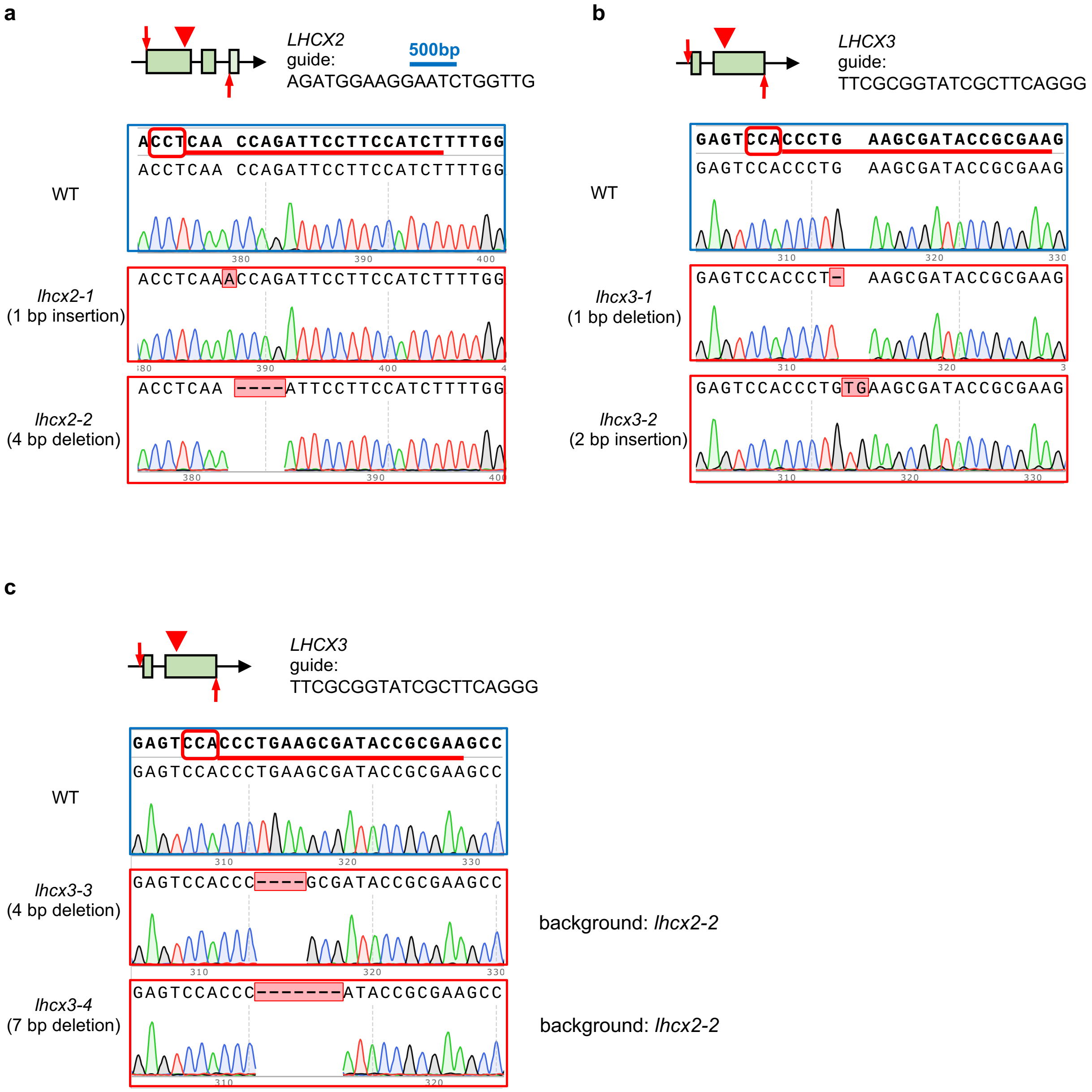

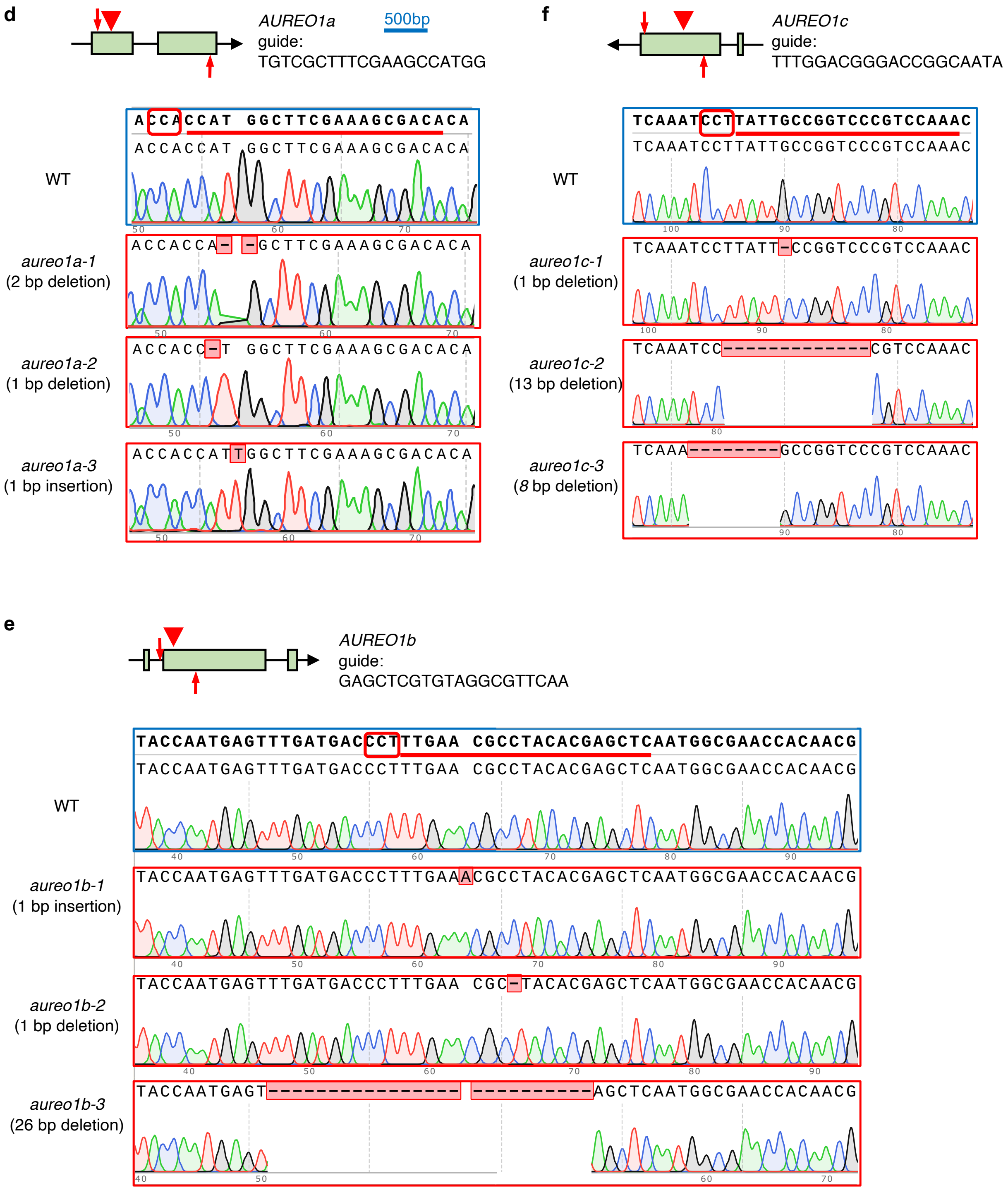

Supplementary Fig. 4 | Genotypes of mutants used in this research. a and **b,** Genotypes of mutants disrupted in *LHCX2* or *LHCX3.* **c,** The double mutants were generated by knocking out *LHCX3* in the *lhcx2-2* background. **d-f,** Genotypes of mutants disrupted in *AUREO1a, AUREO1b* or *AUREO1c*. Open green boxes denote exons; red triangles indicate target sites for

CRISPR/Cas9-mediated mutagenesis. The target sequences are shown above the WT sequence. The protospacer and the protospacer-adjacent motifs or their complementary sequences are labeled with a red line and a red box respectively. PCR was conducted on each strain with primer locations indicated by red arrows (**Supplementary Data 1**). Sequencing was performed using one of the PCR primers and the chromatograms were aligned to the wild-type sequence. For panels **a, b, c, d** and **e**, the sequences are positive strand; for panel **f**, the sequence is negative strand.

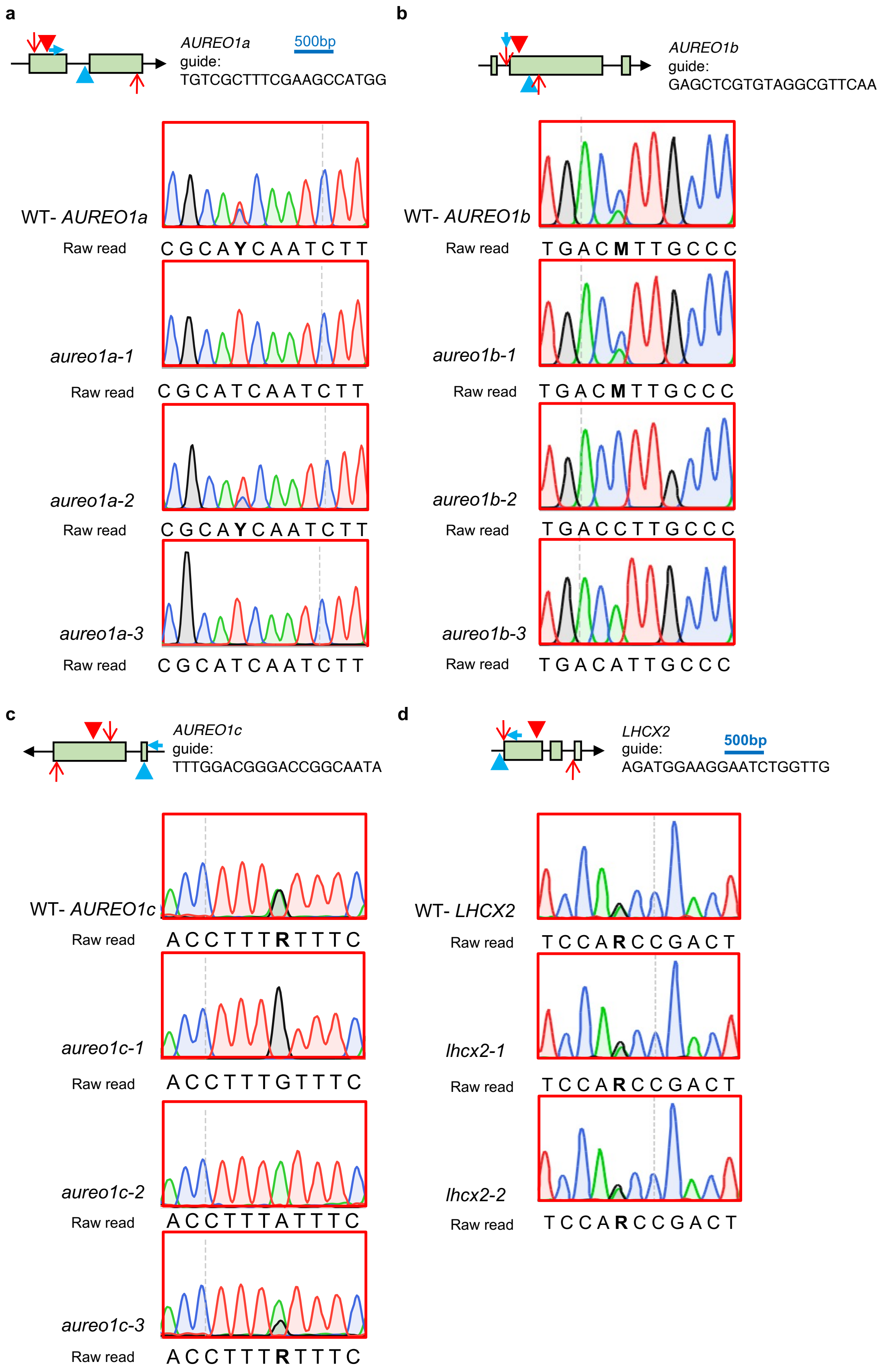

Supplementary Fig. 5 | Single-nucleotide polymorphism (SNP) sequencing results

of PCR products from wild type and mutant lines of *P. tricornutum* used in this study. The mutant lines are for these four genes: *AUREO1a* (**a**), *AUREO1b* (**b**), *AUREO1c* (**c**), and *LHCX2* (**d**). Mutants of *LHCX3* were not investigated because of the lack of SNPs from 1.3 kb upstream to 1.6 kb downstream of the gene in our background strain. For *AUREO1b*, because the SNP concerned is close to the guide region, the sequence presented here is a portion of the same sequencing chromatogram from **Supplementary Fig. 4e**. The detection of polymorphism in *aureo1b-1* substantiates the amplification of both chromosomes. For the other three genes, a PCR (see **Supplementary Data 1** for primer sequences) was performed to encompass both upstream and downstream regions in conjunction with the gene itself. The sequencing primers, highlighted as blue arrows, were designed proximate to an SNP to generate the depicted chromatograms. On the gene models, blue asterisks mark the location of SNPs; red triangles designate target sites for CRISPR/Cas9-mediated mutagenesis, as shown in **Supplementary Fig. 4**. In the chromatograms, bold letters denote SNPs: Y stands for C/T; M for A/C; and R represents A/G.

Supplementary Fig. 6 (to be continued)

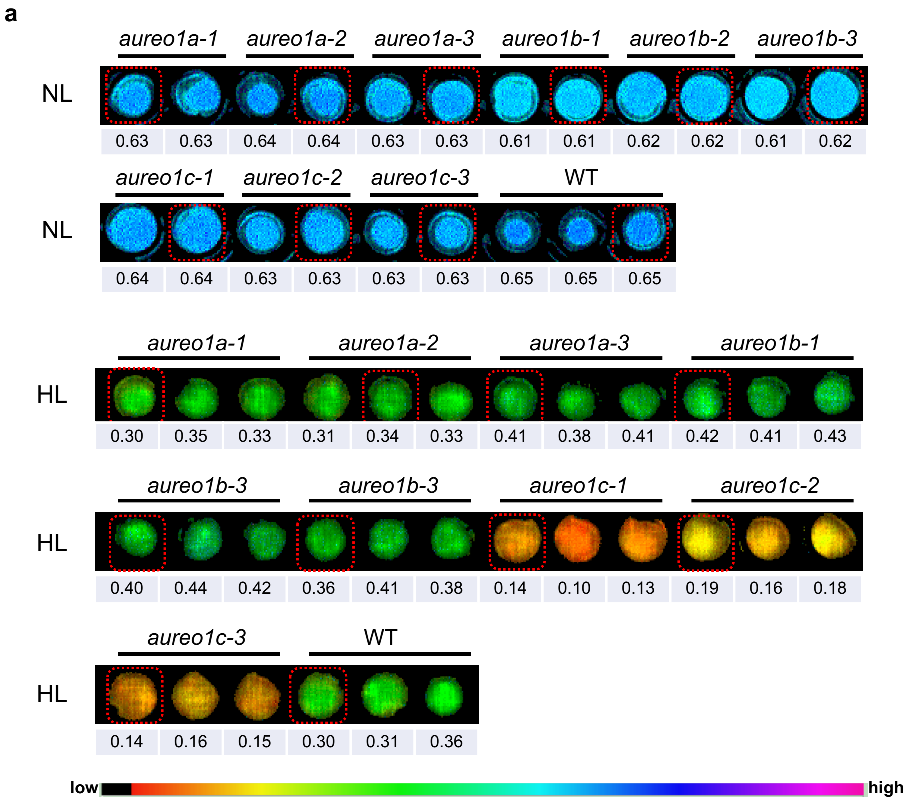

For Fig. 1c

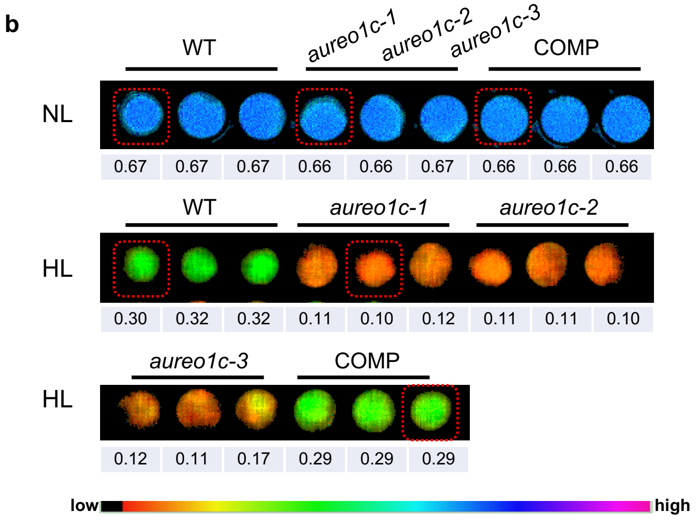

For Fig. 1g

c

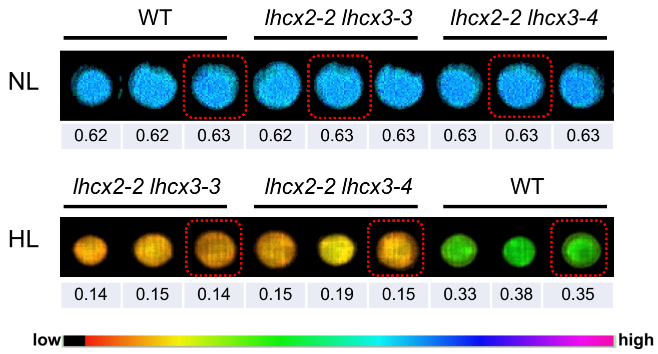

For Fig. 4f

d

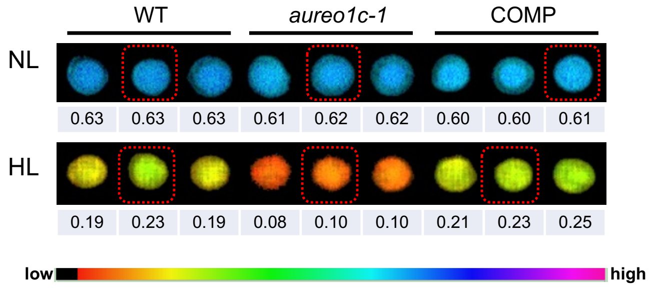

For Fig. 4g

Supplementary Fig. 6 | Uncropped images of the chlorophyll fluorescence phenotypes in main figures, containing data for independent cultures of the same line or data for independent lines. Panels **a to d** correspond to **Fig. 1c, Fig. 1g, Fig. 4f** and **Fig. 4g** respectively. F_v/F_m ratios are shown as a false color image, with values underneath and the color bar at the bottom.

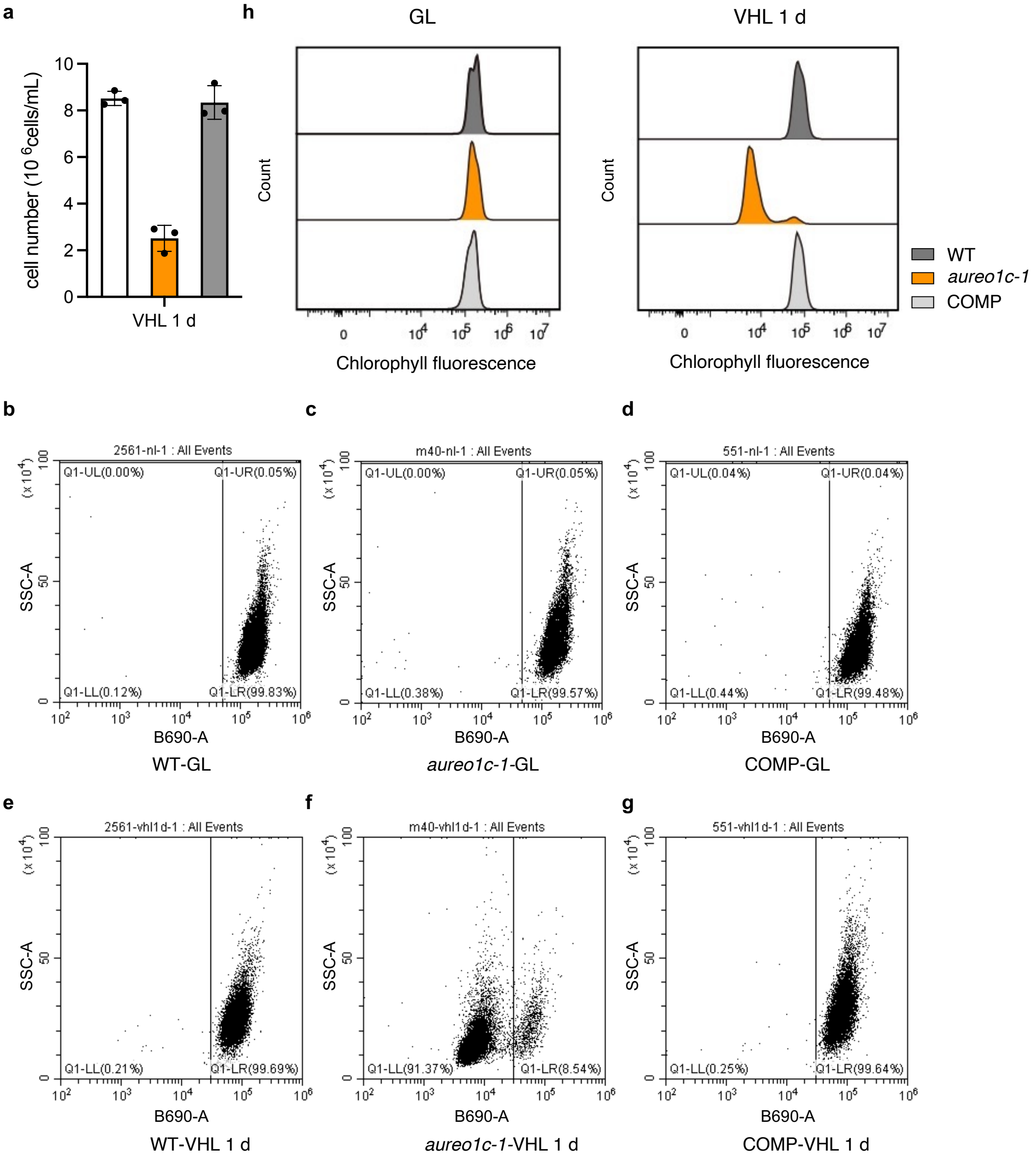

Supplementary Fig. 7 | Cell concentration and chlorophyll fluorescence of wild-type (WT), *aureo1c-1* and COMP cultures after 1 d of very high light (VHL; 1200 μmol photons m^{-2} s^{-1} of white light) treatment. GL, growth light. **a,** each culture was adjusted to a concentration of 4 × 10^{6} cells per mL before the VHL treatment and measured in cell concentration after 1 d of VHL treatment. Data are presented as mean values ± SD. **b-g**, gating strategies for analyzing chlorophyll fluorescence in samples

from GL and VHL (1 d treatment) conditions. **h,** Flow cytometry analysis of cellular chlorophyll fluorescence shown as histograms. The experiments were repeated three times independently with similar results and a representative result is shown. Source data are provided as a Source Data file.

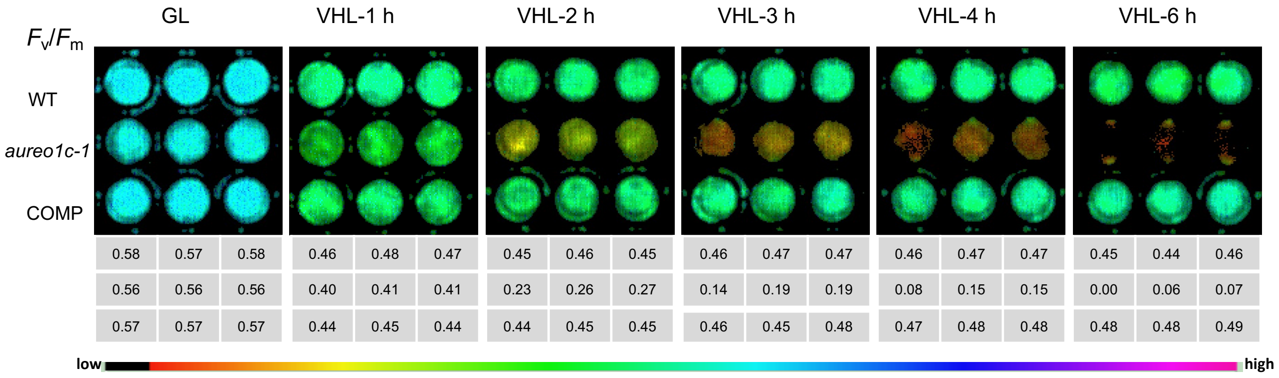

	GL			VHL-1 h			VHL-2 h			VHL-3 h			VHL-4 h			VHL-6 h		
WT	0.58	0.57	0.58	0.46	0.48	0.47	0.45	0.46	0.45	0.46	0.47	0.47	0.46	0.47	0.47	0.45	0.44	0.46
aureo1c-1	0.56	0.56	0.56	0.40	0.41	0.41	0.23	0.26	0.27	0.14	0.19	0.19	0.08	0.15	0.15	0.00	0.06	0.07
COMP	0.57	0.57	0.57	0.44	0.45	0.44	0.44	0.45	0.45	0.46	0.45	0.48	0.47	0.48	0.48	0.48	0.48	0.49

Supplementary Fig. 8 | F_v/F_m measurements of wild-type, *aureo1c-1* and COMP cells during a time course of very high light (VHL; 1200 µmol photons m^{-2} s^{-1} of white light) treatment. GL, growth light. The experiment was repeated three times independently with similar results and a representative result is shown.

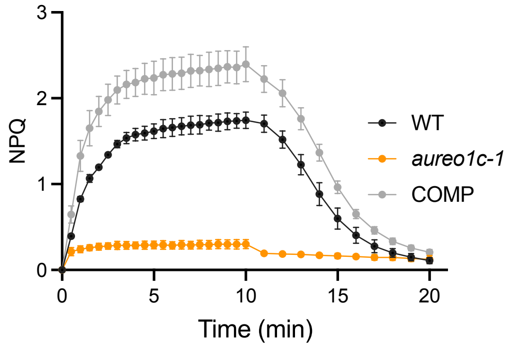

Supplementary Fig. 9 | NPQ measurements of wild-type, *aureo1c-1* and COMP cells after 4 hours of very high light (VHL; 1200 µmol photons m^{-2} s^{-1} of white light) treatment. Three independent cultures were used for the quantification. Data are presented as mean values ± SD. The experiment was repeated three times independently with similar results and a representative result is shown. Source data are provided as a Source Data file.

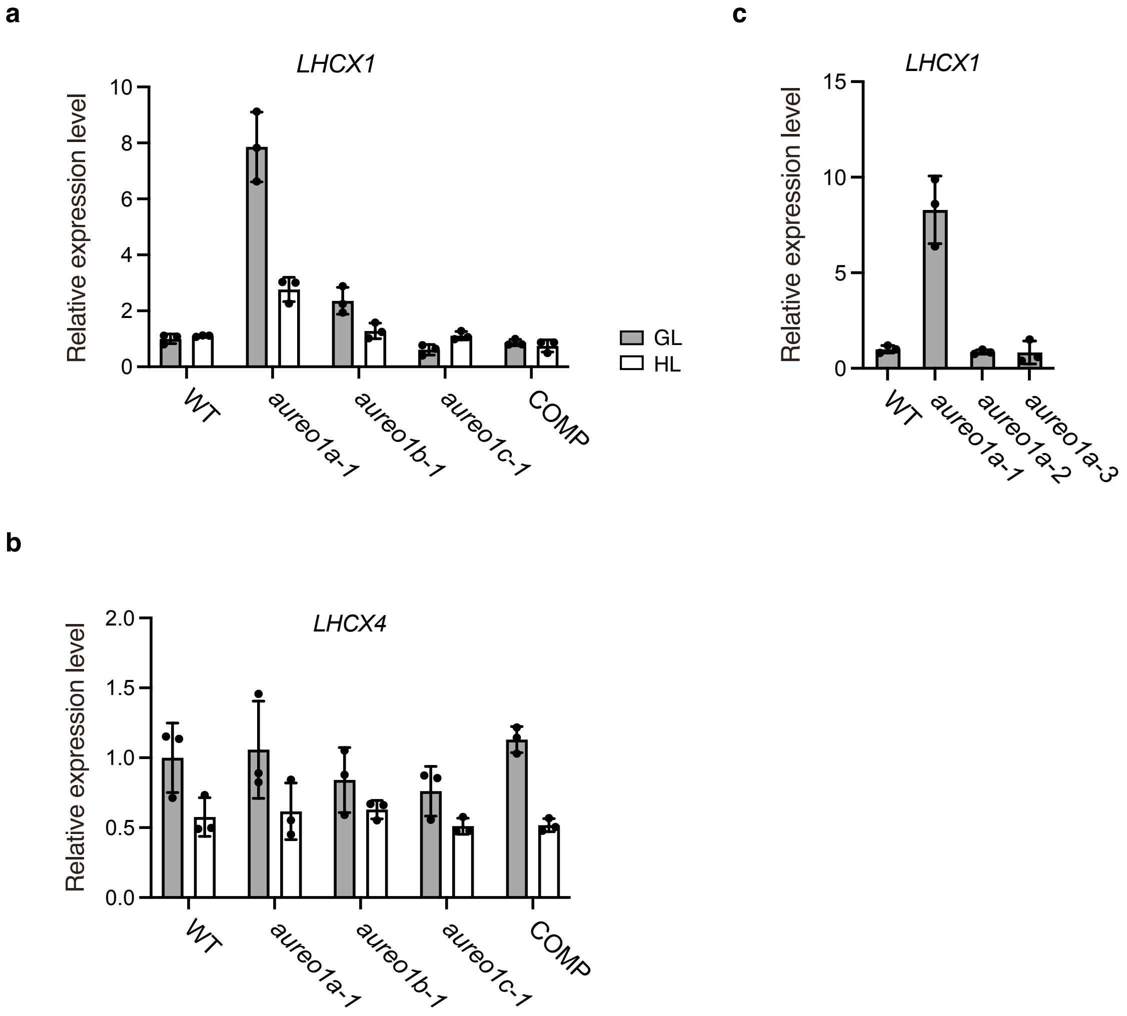

Supplementary Fig. 10 | The effect of aureochrome mutation on the expression of *LHCX1* and *LHCX4* under growth light (GL) and high light (HL, 550 μmol photons m^{-2} s^{-1}) conditions, based on RT-qPCR. a and **b,** The expression of *LHCX1* and *LHCX4* in aureochrome mutants and *aureo1c-1* complementary lines in GL and HL conditions. **c,** The expression of *LHCX1* in wild type and 3 independent *aureo1a* mutant lines in GL condition. For each line, three independent cultures were used for the quantification. Data are presented as mean values ± SD. The experiment was repeated three times independently with similar results and a representative result is shown. Note that although *aureo1a-1* showed a higher level of *LHCX1* expression than wild type under GL, the other two mutant lines of AUREO1a did not. Thus, this phenotype of *aureo1a-1* could be due to other mutations instead of the disruption of

AUREO1a, e.g., off-target effects of CRISPR/Cas9. Source data are provided as a Source Data file.

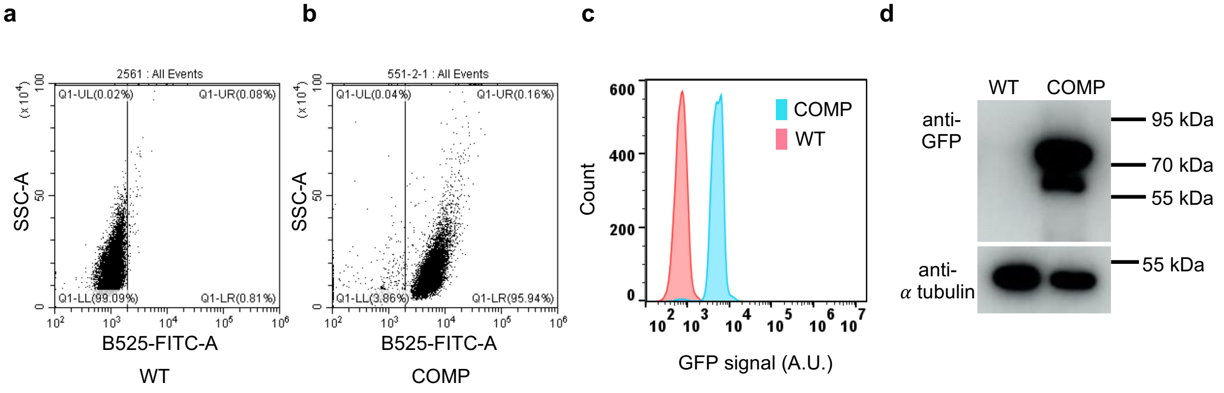

Supplementary Fig. 11 | The COMP line accumulates the AUREO1c-GFP fusion protein. a and **b,** gating strategy to identify GFP-positive cells. **c,** Flow cytometry histogram of the cellular GFP fluorescence in WT and COMP samples. The WT and COMP cells were analyzed using the same parameter settings and gating strategies, and 10000 events were collected. A.U., arbitrary units. **d,** Immunoblotting using antibodies against GFP showing the accumulation of the AUREO1c-GFP fusion protein in the COMP strain. The expected size of AUREO1c-GFP is 63 kDa. Immunoblotting against α-tubulin was performed as a loading control. All the experiments were repeated three times independently with similar results and a representative result is shown. Source data are provided as a Source Data file.

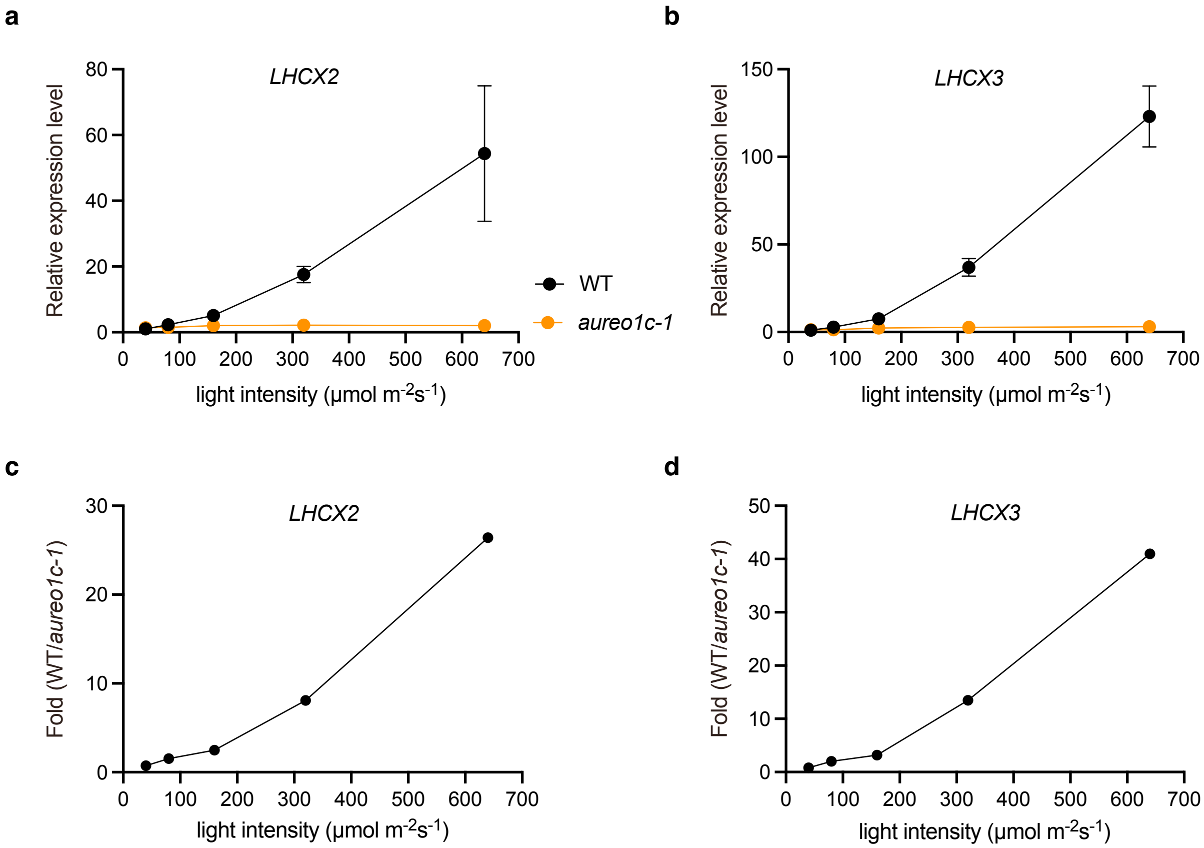

Supplementary Fig. 12 | The induction of *LHCX2/3* is responsive to changes in light intensity and dependent on the presence of AUREO1c. Wild-type and *aureo1c-1* cells were transferred from GL (40 µmol photons m^{-2} s^{-1}) to higher light intensity (80, 160, 320 and 640 µmol photons m^{-2} s^{-1} of white light) for 15 min, and RT-qPCR was performed to measure the transcript abundances of *LHCX2/3*. **a** and **b,** The expression of *LHCX2* and *LHCX3* under different light intensity conditions in wild type and *aureo1c-1* mutant. The wild-type expression level under GL was defined as 1. Three independent cultures were used for the quantification. Data are presented as mean values ± SD. The experiment was repeated three times independently with similar results and a representative result is shown. **c** and **d,** The ratios of *LHCX2* and *LHCX3* transcript abundance between wild type and the *aureo1c-1* mutant under the light intensities tested. Source data are provided as a Source Data file.

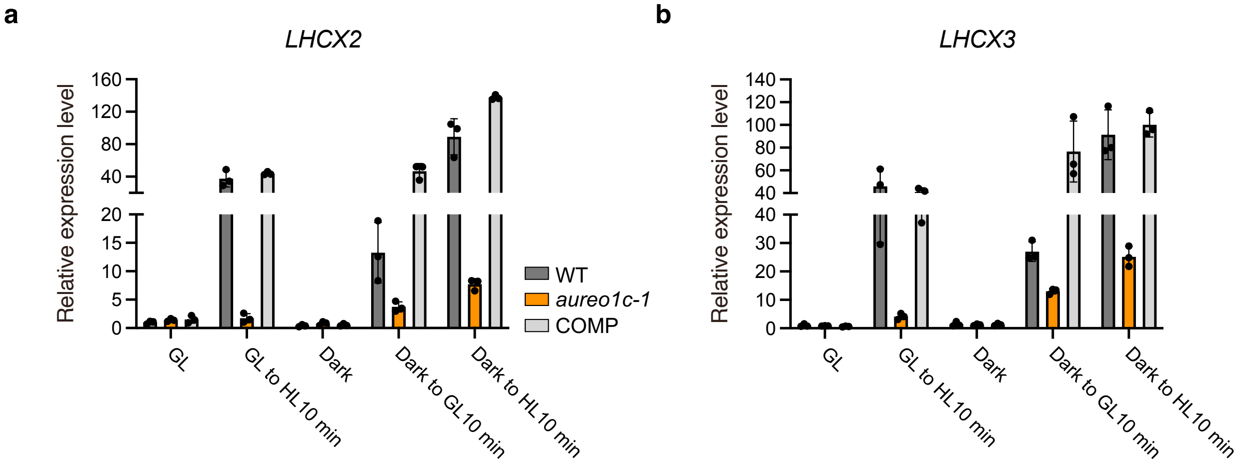

Supplementary Fig. 13 | The transcript of *LHCX2/3* in *P. tricornutum* under different light conditions measured by RT-qPCR. a and **b,** the expression level of *LHCX2* and *LHCX3* of wild type, *aureo1c-1* and COMP cells in GL (growth light; 40 µmol photons m^{-2} s^{-1}), GL to HL (high light; 550 µmol photons m^{-2} s^{-1}) for 10 min, darkness, dark to GL for 10 min and dark to HL for 10 min. Three independent cultures were used for the quantification. Data are presented as mean values ± SD. The experiment was repeated three times independently with consistent results and a representative result is shown. Source data are provided as a Source Data file.

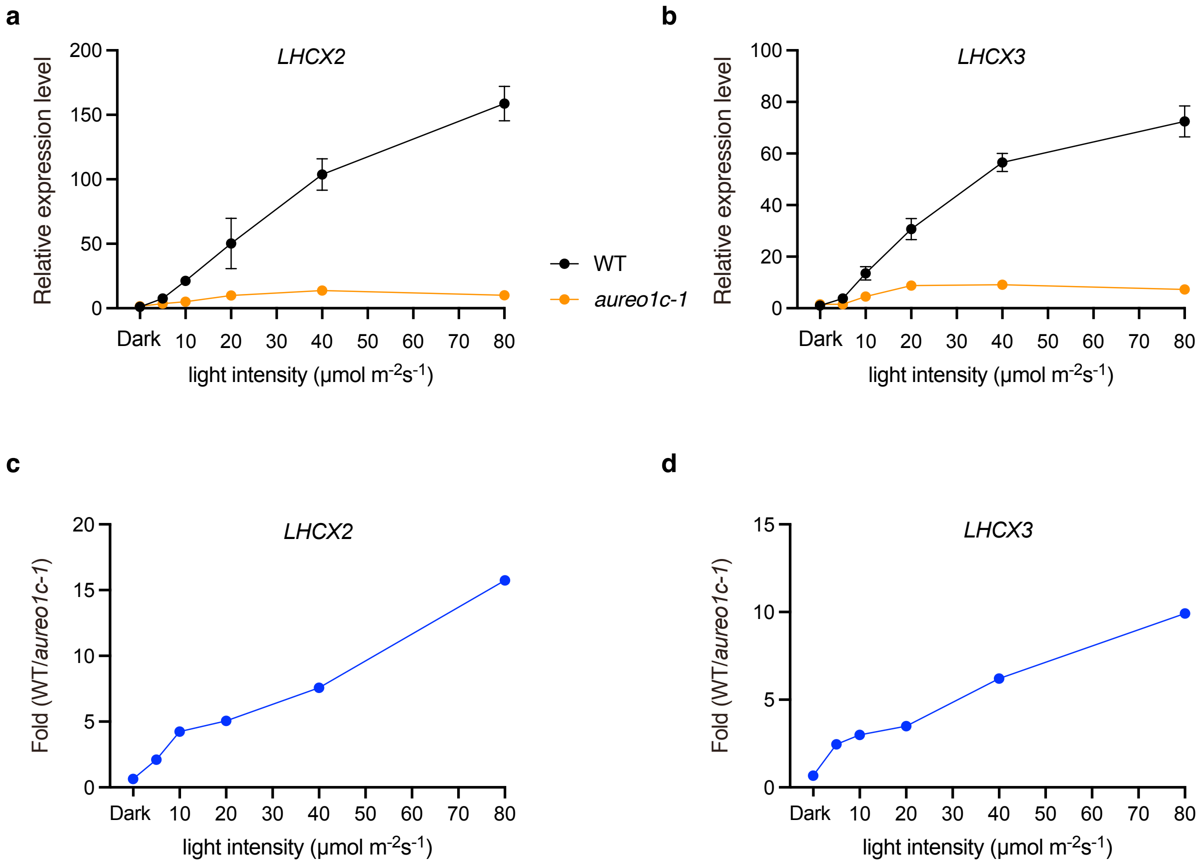

Supplementary Fig. 14 | The induction of *LHCX2/3* is responsive to changes in blue light intensity and dependent on the presence of AUREO1c. Wild-type and *aureo1c-1* cells were transferred from darkness to sequential increase in blue light intensities (5, 10, 20, 40 and 80 μmol photons m^{-2} s^{-1}) for 15 min, and RT-qPCR was performed to measure the transcript abundances of *LHCX2/3*. **a** and **b,** the induction of *LHCX2* and *LHCX3* in response to increasing blue light intensities in wild-type and *aureo1c-1* mutant. The wild-type expression level in dark was defined as 1. Three independent cultures were used for the quantification. Data are presented as mean values ± SD. The experiment was repeated three times independently with similar results and a representative result is shown. **c** and **d,** The ratios of *LHCX2* and *LHCX3* transcript abundance between wild type and the *aureo1c-1* mutant under the light intensities tested. Source data are provided as a Source Data file.

a

b

c

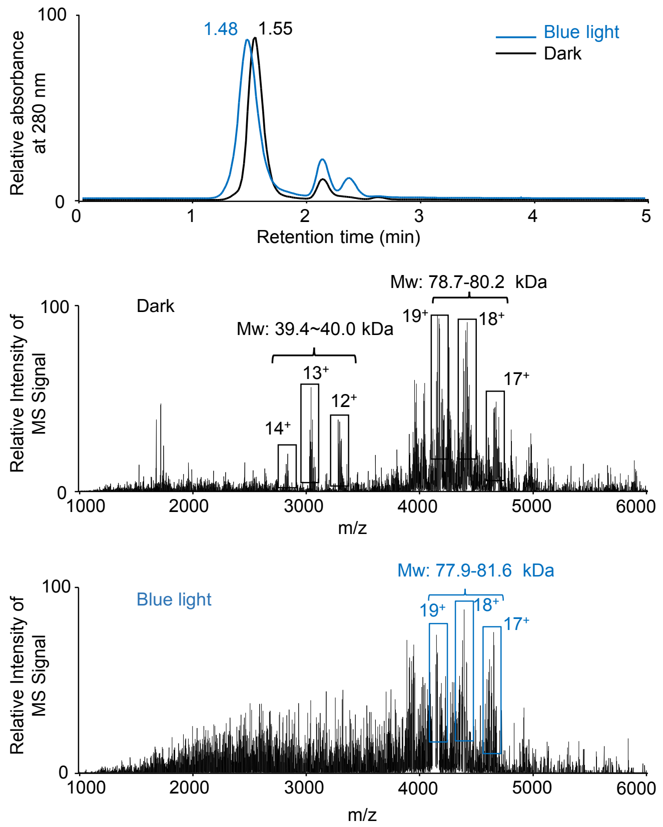

Supplementary Fig. 15 | Liquid chromatography–native mass spectrometry analyses of the recombinant AUREO1c protein. a, Liquid chromatography profiles of recombinant AUREO1c incubated in the dark or in blue light followed by crosslinking. **b** and **c,** Mass spectra of the protein at the peaks of the chromatographic separation. Each cluster of peaks highlighted by rectangles represents a charge state of the ionized protein, and the calculated molecular weight (Mw) range is shown above. The experiment was repeated twice independently with similar results and a representative result is shown.

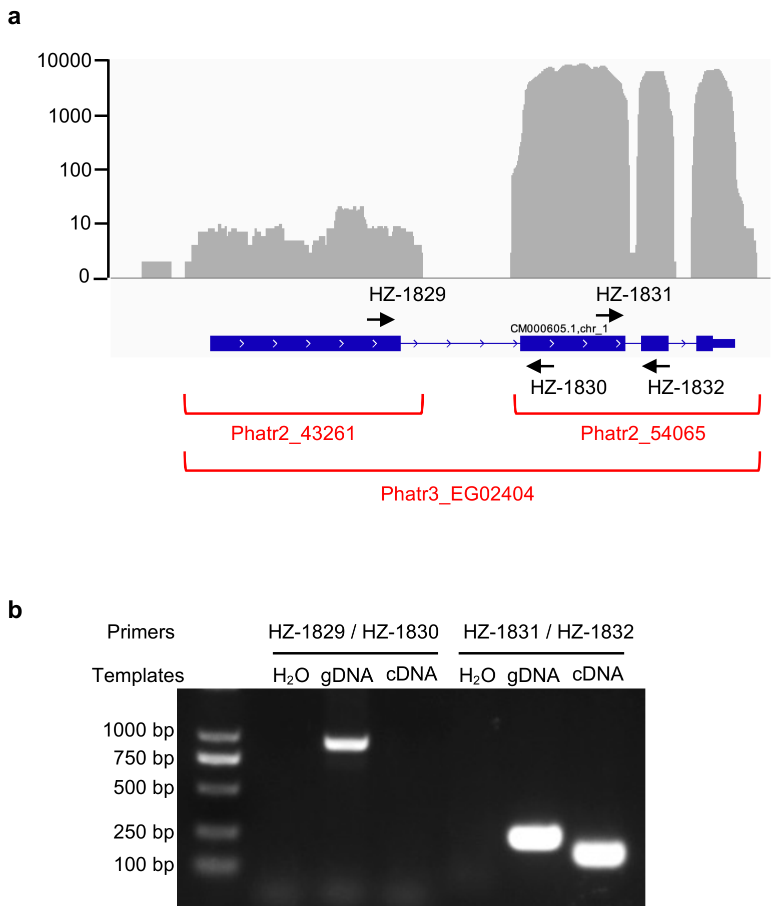

Supplementary Fig. 16 | The Phatr2_54065 model instead of the Phatr3_EG02404 model of *LHCX2* is supported by RNA-Seq results and PCR validation. a, RNA-Seq reads from the HWL condition of wild-type cells in our transcriptome experiment (**Fig. 2**) are displayed in the " linked read view" mode using the Integrative Genomics Viewer (IGV) software. Transcript abundance of regions in the Phatr2_43261 and Phatr2_54065 gene models differed by three orders of magnitude, suggesting that they are two separate genes. **b,** PCRs were performed using genomic DNA (gDNA) and cDNA from wild type as the templates. The lack of product band using the primer pair "HZ-1829/HZ-1830" using cDNA templates suggests that their annealing sites are present in different transcripts. The experiment was repeated three times independently with similar results and a representative result is shown. Source data are provided as a Source Data file.

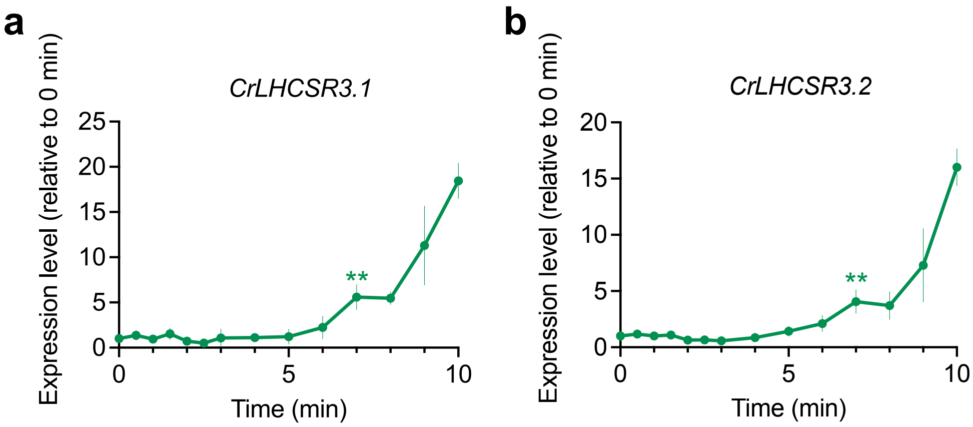

Supplementary Fig. 17 | Transcript abundance of *LHCSR3.1* (a) and *LHCSR3.2* (b) in wild-type *C. reinhardtii* based on RT-qPCR at different time points after the switch from growth light (GL; 40 µmol photons m^{-2} s^{-1} of white light) to high blue light (260 µmol photons m^{-2} s^{-1}). Cells were grown in Tris-acetate-phosphate (TAP) medium both before and during the treatments. Three independent cultures were used for the quantification. Data are presented as mean values ± SD. The experiment was repeated three times independently with similar results and a representative result is shown. The expression levels of *LHCSR3.1/3.2* of wild-type *C. reinhardtii* cells in high blue light treatment were compared with that of GL conditions by a one-sided *t*-test (**, *P* <0.01; the *P* values were 00026 and 0.0051 for *LHCSR3.1* and *LHCSR3.2* respectively). Source data are provided as a Source Data file.

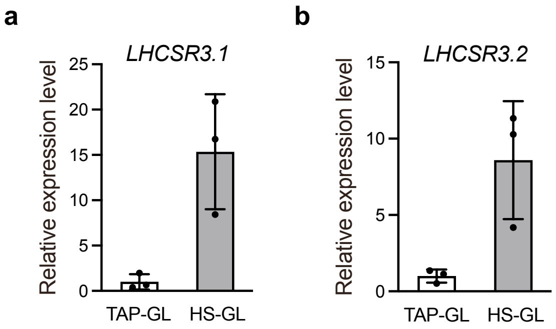

Supplementary Fig. 18 | Transcript abundances of *LHCSR3.1* (a) and *LHCSR3.2* (b) in wild-type *C. reinhardtii* cells cultured in the TAP medium and HS medium respectively, measured by RT-qPCR. GL (growth light): 40 μmol photons m^{-2} s^{-1} of white light. Three independent cultures were used for the quantification. Data are presented as mean values ± SD. The experiment was repeated three times independently with similar results and a representative result is shown. Source data are provided as a Source Data file.

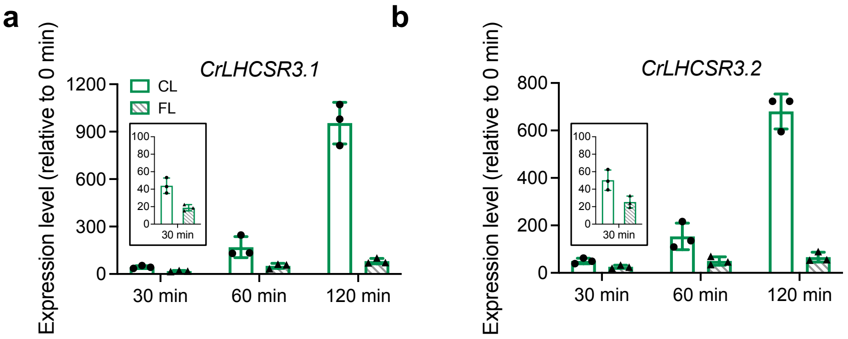

Supplementary Fig. 19 | Transcript abundances of *LHCSR3.1* (a) and *LHCSR3.2* (b) in wild-type *C. reinhardtii*, after CL or FL treatments, relative to those under GL, measured by RT-qPCR. Samples from each condition receiving the same total length of high light treatment were compared. Cells were grown in TAP medium both before and during the treatments. Three independent cultures were used for the quantification. Data are presented as mean values ± SD. The experiment was repeated three times independently with similar results and a representative result is shown. Source data are provided as a Source Data file.

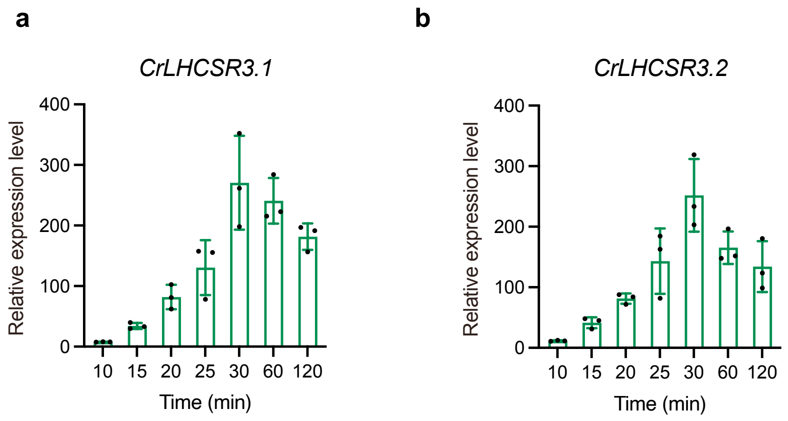

Supplementary Fig. 20 | Transcript abundances of *LHCSR3.1* (a) and *LHCSR3.2* (b) in wild-type *C. reinhardtii*, after continuous high light (CL) treatments, relative to those under GL, measured by RT-qPCR. The cells were cultivated in TAP medium and incubated in HS medium overnight before the CL treatment. Three independent cultures were used for the quantification. Data are presented as mean values ± SD. The experiment was repeated twice independently with similar results and a representative result is shown. This experiment serves as an independent replication of the *C. reinhardtii* CL samples depicted in **Fig. 4c**, with the addition of a more detailed analysis conducted at multiple time points. Source data are provided as a Source Data file.

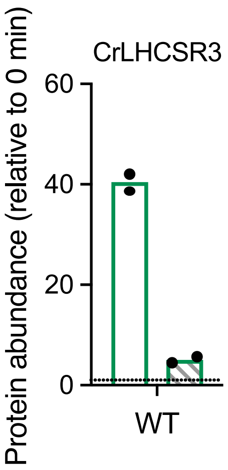

Supplementary Fig. 21 | LHCSR3 protein abundance in *C. reinhardtii* wild type (WT) measured by proteomics. The data were normalized to the protein abundance of LHCSR3 under GL (shown as a dashed line). Cells were grown in TAP medium both before and during the treatments. Two independent cultures were used for the quantification and the means are presented. The experiment was repeated twice independently with similar results and a representative result is shown. Source data are provided as a Source Data file.
