# Supplementary material for: A rapid aureochrome opto-switch enables diatom acclimation to dynamic light
Source: Nat Commun. 2024 Jul 3;15:5578. doi: 10.1038/s41467-024-49991-7 (PMC11219949; doi:10.1038/s41467-024-49991-7)
Supplement: Supplementary file 3 — Description of Additional Supplementary Files [file 41467_2024_49991_MOESM3_ESM.pdf]

Description of Additional Supplementary Files

Supplementary Data 1. Sequences of oligonucleotide primers used in this study.

Supplementary Data 2. Light intensity and quality for each experiment.

Supplementary Data 3. Raw read counts of genes in the RNA-Seq of *P. tricornutum* wild type and the *aureo1c-1* mutant under various light conditions.

Supplementary Data 4. Normalized read counts of genes in the RNA-Seq of *P. tricornutum* wild type and the *aureo1c-1* mutant under various light conditions.

Supplementary Data 5. Differences in the expression levels of each gene between *P. tricornutum* wild type and the *aureo1c-1* mutant under various light conditions and their statistical confidence.

Supplementary Data 6. Gene ontology (GO) term enrichment analysis among AUREO1c-dependent and AUREO1c-independent genes that are regulated in wild type from normal growth light (GL) to high white light (HWL).

Supplementary Data 7. Whole-cell proteomics data for wild-type *C. reinhardtii* (TAP medium) containing relative abundance of each detected protein.

Supplementary Data 8. Whole-cell proteomics data for wild-type *C. reinhardtii* (HS medium) containing relative abundance of each detected protein.

Supplementary Data 9. Whole-cell proteomics data for wild-type *P. tricornutum* and the *aureo1c-1* mutant containing relative abundance of each detected protein.
