# Supplementary material for: A rapid aureochrome opto-switch enables diatom acclimation to dynamic light
Source: Nat Commun. 2024 Jul 3;15:5578. doi: 10.1038/s41467-024-49991-7 (PMC11219949; doi:10.1038/s41467-024-49991-7)
Supplement: Supplementary file 5 — Reporting Summary [file 41467_2024_49991_MOESM5_ESM.pdf]

nature portfolio

Corresponding author(s): Xiaobo Li

Last updated by author(s): Jun 20, 2024

# Reporting Summary

Nature Portfolio wishes to improve the reproducibility of the work that we publish. This form provides structure for consistency and transparency in reporting. For further information on Nature Portfolio policies, see our Editorial Policies and the Editorial Policy Checklist.

## Statistics

For all statistical analyses, confirm that the following items are present in the figure legend, table legend, main text, or Methods section.

| n/a | Confirmed | |
|---|---|---|
| ☐ | ☒ | The exact sample size (*n*) for each experimental group/condition, given as a discrete number and unit of measurement |
| ☐ | ☒ | A statement on whether measurements were taken from distinct samples or whether the same sample was measured repeatedly |
| ☐ | ☒ | The statistical test(s) used AND whether they are one- or two-sided<br>*Only common tests should be described solely by name; describe more complex techniques in the Methods section.* |
| ☐ | ☒ | A description of all covariates tested |
| ☐ | ☒ | A description of any assumptions or corrections, such as tests of normality and adjustment for multiple comparisons |
| ☐ | ☒ | A full description of the statistical parameters including central tendency (e.g. means) or other basic estimates (e.g. regression coefficient) AND variation (e.g. standard deviation) or associated estimates of uncertainty (e.g. confidence intervals) |
| ☐ | ☒ | For null hypothesis testing, the test statistic (e.g. *F*, *t*, *r*) with confidence intervals, effect sizes, degrees of freedom and *P* value noted<br>*Give P values as exact values whenever suitable.* |
| ☒ | ☐ | For Bayesian analysis, information on the choice of priors and Markov chain Monte Carlo settings |
| ☒ | ☐ | For hierarchical and complex designs, identification of the appropriate level for tests and full reporting of outcomes |
| ☐ | ☒ | Estimates of effect sizes (e.g. Cohen's *d*, Pearson's *r*), indicating how they were calculated |

*Our web collection on statistics for biologists contains articles on many of the points above.*

## Software and code

Policy information about availability of computer code

| | |
|---|---|
| Data collection | Walz ImagingWin v2.47 and Walz Dual PAM v1.19 were used for collection of the chlorophyll fluorescence data. |
| Data analysis | Graphpad Prism v8.0 was used for data plotting. DESeq2 was used to perform readcount normalization for RNA-Seq and principal component analysis. ClusterProfiler v4.4.4 was used for determination of the enrichment of gene ontology terms among specific gene sets. |

For manuscripts utilizing custom algorithms or software that are central to the research but not yet described in published literature, software must be made available to editors and reviewers. We strongly encourage code deposition in a community repository (e.g. GitHub). See the Nature Portfolio guidelines for submitting code & software for further information.

## Data

Policy information about availability of data

All manuscripts must include a data availability statement. This statement should provide the following information, where applicable:

- Accession codes, unique identifiers, or web links for publicly available datasets
- A description of any restrictions on data availability
- For clinical datasets or third party data, please ensure that the statement adheres to our policy

For P. tricornutum genes, Phatr3 IDs of the ASM15095v2 genome assembly are provided as follows. AUREO1a: Phatr3_J8113; AUREO1b: Phatr3_J15977; AUREO1c: Phatr3_J51933; LHCX2: Phatr3_EG02404 (PHATRDRAFT_54065); LHCX3: Phatr3_J44733; RPS: Phatr3_J10847; EF1α: Phatr3_J18475; α tubulin: Phatr3_J54534. For C. reinhardtii genes, v5.6 IDs of the ABCN02000000 genome assembly are provided as follows. LHCSR3.1: Cre08.g367500; LHCSR3.2: Cre08.g367400; RACK1, Cre06.g278222.

Raw transcriptome files from the sequencing platform have been uploaded to NCBI Sequence Read Archive (SRA; https://www.ncbi.nlm.nih.gov/sra). SRA Accession number are SAMN34393917, SAMN34393918, SAMN34393919 (wild type in growth light); SAMN34393932, SAMN34393933, SAMN34393934 (aureo1c-1 in growth light); SAMN34393911, SAMN34393912, SAMN34393913 (wild type in high red light); SAMN34393926, SAMN34393927, SAMN34393928 (aureo1c-1 in high red light); SAMN34393914, SAMN34393915, SAMN34393916 (wild type in high white light); SAMN34393929, SAMN34393930, SAMN34393931 (aureo1c-1 in high white light); SAMN34393905, SAMN34393906, SAMN34393907 (wild type in high blue light 1); SAMN34393920, SAMN34393921, SAMN34393922 (aureo1c-1 in high blue light 1); SAMN34393908, SAMN34393909, SAMN34393910 (wild type in high blue light 2); SAMN34393923, SAMN34393924, SAMN34393925 (aureo1c-1 in high blue light 2). The RNA-seq data was related to Fig. 2b-d and Fig. 3a-d. Accession numbers for each individual transcriptome are also provided in Supplementary Data 3.

The mass spectrometry proteomics data have been deposited to the ProteomeXchange Consortium via the PRIDE partner repository with the dataset identifier PXD045342 for Phaeodactylum tricornutum, PXD045344 for Chlamydomonas reinhardtii carried out in TAP medium, and PXD050435 for Chlamydomonas reinhardtii carried out in HS medium. The proteomic data was related to Fig. 4d and Supplementary Fig. 21.

The SRA Accession numbers of DAP-seq data in NCBI are SAMN37389769 (incubating AUREO1c-GST fusion protein with DNA in vitro) and SAMN37389768 (incubating GST protein with DNA in vitro, control) and related to Fig. 3e.

## Research involving human participants, their data, or biological material

Policy information about studies with human participants or human data. See also policy information about sex, gender (identity/presentation), and sexual orientation and race, ethnicity and racism.

| | |
|---|---|
| Reporting on sex and gender | *Use the terms sex (biological attribute) and gender (shaped by social and cultural circumstances) carefully in order to avoid confusing both terms. Indicate if findings apply to only one sex or gender; describe whether sex and gender were considered in study design; whether sex and/or gender was determined based on self-reporting or assigned and methods used. Provide in the source data disaggregated sex and gender data, where this information has been collected, and if consent has been obtained for sharing of individual-level data; provide overall numbers in this Reporting Summary. Please state if this information has not been collected. Report sex- and gender-based analyses where performed, justify reasons for lack of sex- and gender-based analysis.* |
| Reporting on race, ethnicity, or other socially relevant groupings | *Please specify the socially constructed or socially relevant categorization variable(s) used in your manuscript and explain why they were used. Please note that such variables should not be used as proxies for other socially constructed/relevant variables (for example, race or ethnicity should not be used as a proxy for socioeconomic status). Provide clear definitions of the relevant terms used, how they were provided (by the participants/respondents, the researchers, or third parties), and the method(s) used to classify people into the different categories (e.g. self-report, census or administrative data, social media data, etc.) Please provide details about how you controlled for confounding variables in your analyses.* |
| Population characteristics | *Describe the covariate-relevant population characteristics of the human research participants (e.g. age, genotypic information, past and current diagnosis and treatment categories). If you filled out the behavioural & social sciences study design questions and have nothing to add here, write "See above."* |
| Recruitment | *Describe how participants were recruited. Outline any potential self-selection bias or other biases that may be present and how these are likely to impact results.* |
| Ethics oversight | *Identify the organization(s) that approved the study protocol.* |

Note that full information on the approval of the study protocol must also be provided in the manuscript.

# Field-specific reporting

Please select the one below that is the best fit for your research. If you are not sure, read the appropriate sections before making your selection.

[x] Life sciences [ ] Behavioural & social sciences [ ] Ecological, evolutionary & environmental sciences

For a reference copy of the document with all sections, see nature.com/documents/nr-reporting-summary-flat.pdf

# Life sciences study design

All studies must disclose on these points even when the disclosure is negative.

| | |
|---|---|
| Sample size | We used triplicates for gene expression analyses or physiological parameter analyses, and duplicates for proteomic measurements. These are commonly used sample sizes for the corresponding experiment. As specified in the legend of each figure, we always have independent sets of replicates beyond the experiments plotted and the results are consistent. |
| Data exclusions | No data were excluded. |
| Replication | All attempts at replication were successful. |
| Randomization | For each algal strain involved in this study, each time three cultures were analyzed at the same time in parallel and randomization is not relevant in these cases. |

Blinding | We did not do blinding because it is not a common practice in the types of experiments that we performed.

# Reporting for specific materials, systems and methods

We require information from authors about some types of materials, experimental systems and methods used in many studies. Here, indicate whether each material, system or method listed is relevant to your study. If you are not sure if a list item applies to your research, read the appropriate section before selecting a response.

## Materials & experimental systems

| n/a | Involved in the study |
|---|---|
| ☐ | ☒ Antibodies |
| ☐ | ☒ Eukaryotic cell lines |
| ☒ | ☐ Palaeontology and archaeology |
| ☒ | ☐ Animals and other organisms |
| ☒ | ☐ Clinical data |
| ☒ | ☐ Dual use research of concern |
| ☒ | ☐ Plants |

## Methods

| n/a | Involved in the study |
|---|---|
| ☒ | ☐ ChIP-seq |
| ☐ | ☒ Flow cytometry |
| ☒ | ☐ MRI-based neuroimaging |

## Antibodies

Antibodies used | Goat Anti-Venus/GFP (SICGEN AB2166, 1: 2000 dilution). Rabbit anti α-Tubulin (Beyotime AF5012, 1: 2000 dilution) .

Validation | Both antibodies are from commercial sources. For SICGEN AB2166, the manufacturer stated "This antibody also recognizes Venus/GFP and does not cross-react to mCherry/red fluorescent proteins." For Beyotime AF5012, the manufacturer's website showed specific blotting results against α-Tubulin in multiple eukaryotic cell lines.

## Eukaryotic cell lines

Policy information about cell lines and Sex and Gender in Research

Cell line source(s) | The wild-type Phaeodactylum tricornutum (P. tricornutum) strain CCMP2561 was acquired from The National Center for Marine Algae and Microbiota (https://ncma.bigelow.org/). Mutant strains were generated in our lab from this background.

Authentication | *Describe the authentication procedures for each cell line used OR declare that none of the cell lines used were authenticated.*

Mycoplasma contamination | *Confirm that all cell lines tested negative for mycoplasma contamination OR describe the results of the testing for mycoplasma contamination OR declare that the cell lines were not tested for mycoplasma contamination.*

Commonly misidentified lines (See ICLAC register) | *Name any commonly misidentified cell lines used in the study and provide a rationale for their use.*

## Flow Cytometry

### Plots

Confirm that:

☒ The axis labels state the marker and fluorochrome used (e.g. CD4-FITC).

☒ The axis scales are clearly visible. Include numbers along axes only for bottom left plot of group (a 'group' is an analysis of identical markers).

☒ All plots are contour plots with outliers or pseudocolor plots.

☒ A numerical value for number of cells or percentage (with statistics) is provided.

### Methodology

Sample preparation | No staining were performed.

Instrument | CytoFLEX LX Flow Cytometer (Beckman Coulter) was used for signal detection.

Software | CytExpert and FlowJo (BD Biosciences) were used to collect and analyze the flow cytometry data respectively.

Cell population abundance | No sorting was performed.

Gating strategy | For Supplementary Figure 11, the gating strategy was precisely defined utilizing the B525-FITC-A fluorescence channel, which closely corresponds to the emission spectrum of GFP. Wild-type cells, which inherently lack GFP fluorescence, were

employed as a baseline to accurately distinguish between GFP-positive and GFP-negative populations. In Supplementary Figure 7, the gating strategy was carefully crafted based on the B690-A channel, selected for its proximity to the characteristic emission wavelength of chlorophyll. This allowed for the effective discrimination of cells based on chlorophyll fluorescence.

☒ Tick this box to confirm that a figure exemplifying the gating strategy is provided in the Supplementary Information.
